# HACE1 is a potential tumor suppressor in osteosarcoma

**DOI:** 10.1038/s41419-018-1276-4

**Published:** 2019-01-08

**Authors:** Amal M El-Naggar, Paul W. Clarkson, Gian Luca Negri, Busra Turgu, Fan Zhang, Michael S. Anglesio, Poul H. Sorensen

**Affiliations:** 1 Department of Pathology and Laboratory Medicine, Faculty of Medicine, University of British Columbia, Vancouver, BC Canada; 2 Department of Molecular Oncology, British Columbia Cancer Research Centre, Vancouver, BC Canada; 3 Department of Pathology, Faculty of Medicine, Menoufia University, Menoufia Governorate, Egypt; 4 Department of Orthopedics, University of British Columbia, Vancouver, British Columbia Canada; 5 Vancouver Prostate Centre, University of British Columbia, Vancouver, British Columbia Canada; 6 Department of Obstetrics and Gynecology, University of British Columbia, Vancouver, British Columbia Canada

## Abstract

Osteosarcoma is a malignant bone sarcoma characterized by extensive genomic disruption and a propensity for metastatic spread. Osteoid production suggests a close relationship with normal osteoblasts, and the latter are the presumptive cell of origin of this disease. The *HACE1* gene, localized to human chromosome 6q21, encodes the HACE1 HECT E3 ligase, a tumor suppressor in diverse tumors that acts in part by targeting the activated form of RAC1 GTPase for proteasomal degradation. Disruption or loss of 6q21 is relatively common in osteosarcomas, and *Hace1−/−/Tp53*+/*−* mice frequently develop osteosarcomas, in contrast to *Tp53*+/− mice, which do not. This suggests an unexplored link between *HACE1* loss and osteosarcoma. Here we compared *HACE1* expression in normal osteoblasts and osteosarcoma cell lines in vitro by western blotting and quantitative RT-PCR, and in human osteosarcoma specimens by immunohistochemistry. Both *HACE1* transcript and protein levels were reduced in osteosarcoma compared to osteoblasts in vitro. Reduced HACE1 expression in osteosarcoma tumors was observed in 76% of cases and associated with high-grade lesions. Further, clonally derived pairs of high and low metastatic osteosarcoma cell lines showed significant downregulation in the high compared to corresponding low metastatic cells. Ectopic expression of HACE1 markedly inhibited anchorage-independent growth and cell motility of HACE1 osteosarcoma cell lines, and was associated with reduced RAC1 activation and decreased reactive oxygen species (ROS). Finally, HACE1 overexpression blocked osteosarcoma xenograft growth and dramatically reduced pulmonary metastases. These findings point to a potential tumor suppressor function for *HACE1* in osteosarcoma.

## Introduction

Osteosarcoma is a malignant bone tumor commonly arising in areas of rapid bone growth, such as the distal femur and proximal tibia^1–3^. It represents the most common bone sarcoma, comprising approximately 20% of all bone tumors and about 5% of pediatric tumors^1^. It is predominantly a disease of adolescence and young adulthood, with 60% of patients aged under 25 years at diagnosis; however, there is a second peak of incidence in later life, with 30% of patients being over 40 years of age^4^. Several sub-types of osteosarcoma are described, which have in common the production of osteoid by malignant cells, and a propensity for metastatic spread, particularly to lungs^2,5^.

Most cases of osteosarcoma are sporadic, but certain environmental and hereditary factors have been associated with elevated risk osteosarcoma^6,7^. The former include exposure to ionizing radiation and Paget disease, with dysregulated bone recycling, both of which well-recognized risk factors for the development of secondary osteosarcoma^8,9^. Hereditary conditions associated with osteosarcoma include familial retinoblastoma, Li–Fraumeni syndrome, and Rothmund–Thomson syndrome^10^. The tumor suppressor gene *TP53* is the most well-characterized gene implicated in osteosarcoma^6^. Loss of p53 due to somatic mutation, or germline inactivation as in the autosomal dominant disorder Li–Fraumeni syndrome, predisposes to osteosarcoma^11–13^. *TP53* is commonly inactivated in osteosarcoma either by allelic loss, point mutations, or gene rearrangements^12,14,15^. Up to 26.5% of non-hereditary osteosarcoma cases show somatic loss of p53^16^, and 30% of Li–Fraumeni syndrome patients develop osteosarcoma^17^. *TP53* mutations are associated with unfavorable outcome^18^, and up to 60% of high-grade osteosarcomas show *TP53* mutations, compared with 1% of low-grade osteosarcoma^11,19,20^.

Another well-characterized gene implicated in osteosarcoma is *RB1*, localized to chromosome 13q14 and encoding the 110 kDa pRB1 protein that negatively regulates cell cycle progression^21^. Loss of RB in osteosarcoma is associated with poor patient outcome^22,23^. Other genetic abnormalities are also associated with increased risk of osteosarcoma, such as mutations of RecQ helicases, germline *RECQL4* inactivation resulting in Rothmund–Thomson syndrome^24^, *BLM*(*RECQL3)* inactivation resulting in Bloom syndrome, or *WRN* inactivation resulting in Werner syndrome^11,25^. MicroRNA and copy number variation (CNV) analyses have further identified hsa-miR-27a-3p, hsa-miR-9-5p, hsa-miR-182-5p, *FRS2*, *CORO1C*, *FOXP1,* and *CPEB4* as potentially contributing to the pathogenesis of osteosarcoma^26^. Moreover, next-generation sequencing of patients with conventional high-grade osteosarcoma identified 15 genes with variations only in the treatment non-responder patients, including *ERBB4*, Thrombospondin I (*THBS1*), *DIS3*, and *BCLAF*^27^. Whole-genome sequencing of 20 osteosarcomas and matched normal tissue showed multiple tumor-associated structural variations and copy number alterations as well as kataegis (single-nucleotide variations with localized hypermutation)^28^. Of note, p53 pathway mutations were identified in all tumor samples. Somatic alterations affecting *RB1, ATRX*, and *DLG2* genes were detected in 29–53% of the tumors. Recently, exome sequencing of 31 osteosarcomas showed that over 80% exhibited mutational signatures characteristic of *BRCA1/2* deficiency^29^, further highlighting the role of altered DNA damage repair pathways in osteosarcoma.

*HACE1* (HECT domain and ankyrin-repeat-containing E3 ubiquitin-protein ligase 1) was originally cloned from chromosome 6q21 translocation breakpoints in pediatric Wilms’ tumor^30^. HACE1 is a HECT family E3 ligase with an N-terminal ankyrin-repeat domain (ANK) that binds substrates for ubiquitylation, and a conserved C-terminal catalytic HECT domain that is responsible for HACE1 ligase activity^30,31^. It was further shown that conserved Cys-876 of the HACE1 HECT domain functions to bind ubiquitin for subsequent transfer to HACE1 substrates^30^. Hace1 targets the activated form of the RAC1 GTPase for ubiquitylation and subsequent proteosomal degradation^32,33^. By targeting RAC1 at membrane-associated RAC1-dependent NADPH oxidase complexes, HACE1 reduces ROS levels in vitro and in vivo by blocking NADPH oxidase-mediated superoxide generation^34^. Recently, it was shown that HACE1 is phosphorylated at serine 385 by PAK1 kinase, resulting in lower efficiency of RAC1 ubiquitination^35^. Further, HACE1 has been shown to play critical roles in TNFR1 signaling^36^. HACE1 is also reported to ubiquitylate the autophagy receptor Optineurin (OPTN), which in turn facilitates OPTN interactions with p62/SQSTM1 to activate autophagy to inhibit growth and tumorigenicity of lung cancer cells^37^. HACE1 also provides cytoprotective regulation of proteotoxic stress responses, such as in cardiac cells^38^. Moreover HACE1, via interactions with Rab proteins, is targeted to Golgi membranes, regulating Golgi biogenesis, Golgi traffic, and postmitotic Golgi membrane fusion^39^.

*HACE1* expression is reduced in many tumor types compared to corresponding normal tissues, including Wilms’ tumor^30,31^, breast carcinoma^40^, lung adenocarcinoma, angiosarcoma, and lymphoma^31^. Moreover, loss of heterozygosity at chromosome 6q21 is described in multiple cancers^31^, including ovarian carcinoma, non-Hodgkin’s lymphoma, pancreatic carcinoma, prostate carcinoma, and osteosarcoma^31,41^. Previously, we reported that *Hace1−/−* mice develop late onset tumors across all three germ layers, including osteosarcomas^31^. Moreover, crossing *Hace1*−/− mice into a *Tp53*+/− background led to an increased rate of osteosarcoma compared to *Hace1−/−* mice, while osteosarcomas were not observed in *Hace1*+/+*/Tp53*+/− mice^31^. This suggests that *HACE1* loss is associated with osteosarcoma development. However, studies directly addressing the link between HACE1 and osteosarcoma are currently lacking.

## Results

### HACE1 expression is reduced in osteosarcoma cells compared to normal osteoblasts

**Fig. 1:**
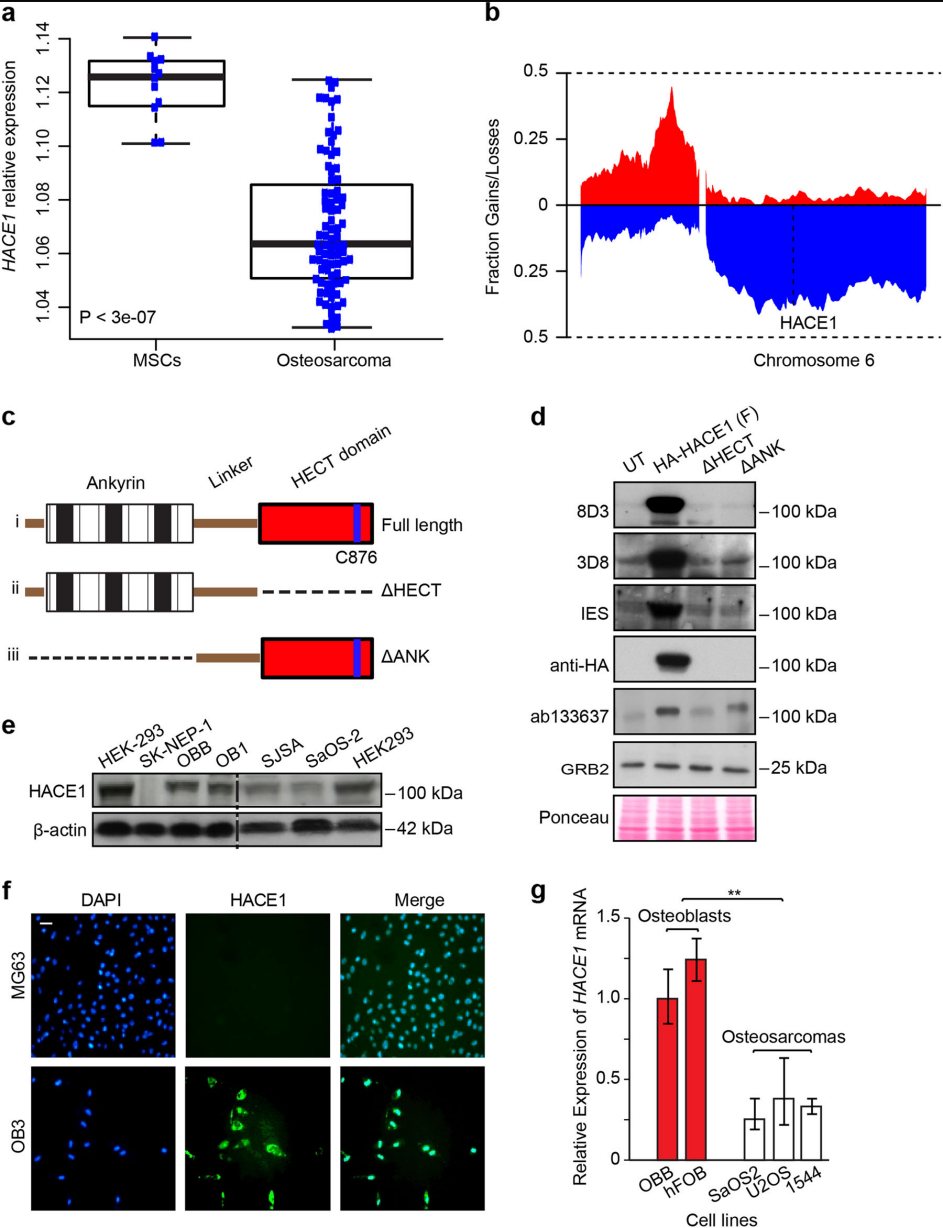
HACE1 expression in osteosarcoma. **a** Relative expression of HACE1 is significantly higher (Wilcoxon test <0.05) in mesenchymal stem cells (12 samples, GSE28974) compared to osteosarcoma tumor biopsies (84 samples, GSE33382). **b** Genome-wide analysis of somatic copy number alterations in osteosarcoma. The plot shows fractions of copy number loss/gain of a cohort of 113 primary osteosarcoma specimens across chromosome 6. **c** Diagrammatic representation of the full-length HACE1 protein structure (i) and mutants lacking the HECT domain (ΔHECT; ii) or the ankyrin repeats (ΔANK; iii) used in this study. **d** OST cells were transfected with the indicated vectors. Immunoblots showing successful transfection of OST cell line with the indicated vectors as detected by the generated clones of HACE1 antibodies described in the Material and methods, and compared to commercially available HACE1 antibody (Abcam, cat # ab133637). GRB2 and Ponceau staining showing equal protein load. **e** Immunoblot showing HACE1 protein expression in osteoblasts and osteosarcoma cell lines. HEK-293 was used as a positive control while SK-NEP-1 served as a negative control for HACE1 expression. β-Actin was used as a loading control. **f** Immunofluorescence analysis of HACE1 expression in MG63 osteosarcoma cell line and OB3 osteoblast cell line displaying strong nucleo-cytoplasmic staining in OB3 cells contrary to remarkably weak cytoplasmic staining in MG63. Blue: DAPI (nuclear stain); green: HACE1. Scale bars, 50 μm. **g** *HACE1* mRNAs expression in osteoblast and osteosarcoma cell lines. The overall relative levels of *HACE1* mRNAs were measured using quantitative RT-PCR in the indicated osteoblasts and osteosarcoma cell lines. PCR reactions were performed twice in quadruplicate. The data were normalized to endogenous β-actin, and presented as mean ± SD. Two-tailed Student's *t*-test was used to analyze the data with **p-value < 0.001

**Fig. 2:**
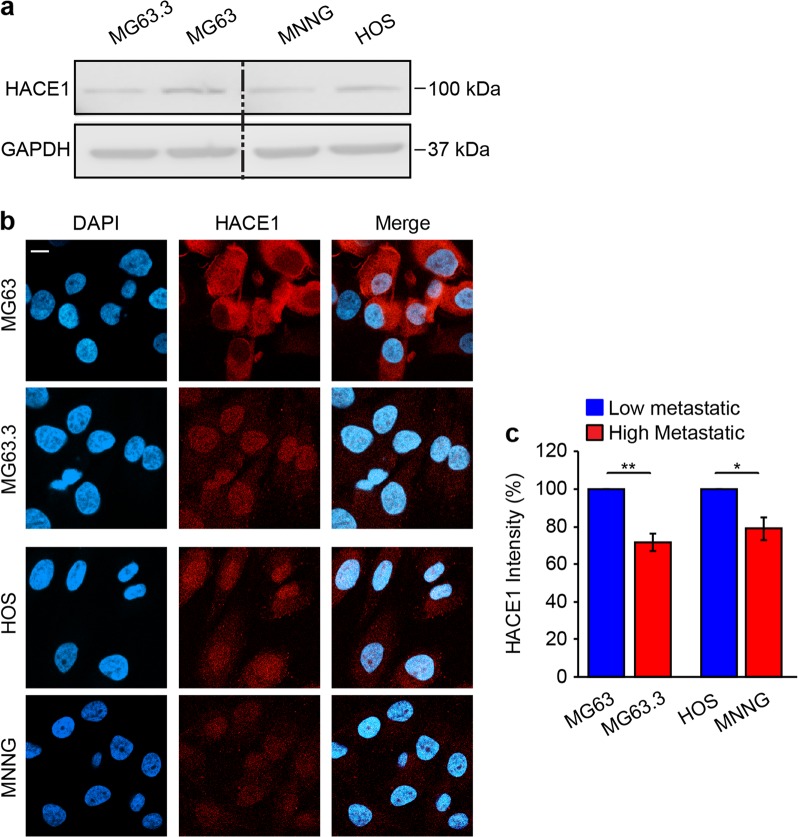
HACE1 expression in high vs low metastatic osteosarcoma cells. **a** Immunoblot showing HACE1 protein expression in clonally derived pairs of high/low metastatic osteosarcoma cells, MG63.3/MG63 and MNNG/HOS. GAPDH was used as a loading control. **b** Immunofluorescence analysis of HACE1 expression in pairs of high/low metastatic MG63.3/MG63 and MNNG/HOS osteosarcoma cell line pairs. Blue: DAPI (nuclear stain); red: HACE1. Scale bars, 10 μm. **c** Quantitation of HACE1 signal intensity in  **b**. Error bars indicate SEM for *n* = 10 HPFs. *p-value < 0.05, **p-value < 0.005

To investigate potential links between *HACE1* and osteosarcoma, we first analyzed publicly available databases for *HACE1* mRNA expression in osteosarcoma samples (GSE33382) compared to mesenchymal stem cells (MSCs) (GSE28974). *HACE1* expression was significantly lower in osteosarcoma samples compared to MSCs (Fig. 1a). Next, to evaluate CNVs affecting chromosome 6, we analyzed publicly available data on 113 fresh-frozen osteosarcoma tissue samples from pretreated osteosarcoma biopsies (E-MTAB-4815; see Materials and methods^42^). This identified copy number losses of the entire long arm of chromosome 6, including the HACE1 locus, in ~36% of patients, further implicating HACE1 deficiency in osteosarcomagenesis (Fig. 1b). Next we assessed HACE1 protein expression in different cell lines using HACE1 antibodies generated in our laboratory that detect full-length HACE1 or HACE1 fragments lacking either the HECT domain or ankyrin repeats, compared with a control commercially available anti-HACE1 antibody (Abcam cat# ab133637), as shown in Fig. 1c–d. HACE1 was highly expressed in previously described osteoblast cell lines (OBB and OB1 (ref. ^43^)) compared to SJSA and SaOS-2 osteosarcoma cell lines (Fig. 1e), while HEK-293 cell line served as a positive control and Ewing sarcoma SK-NEP-1 cells as a negative control for HACE1 expression, as previously reported^30,31^. Moreover, immunofluorescence showed strong staining for HACE1 in normal osteoblasts compared to MG63 osteosarcoma cells, further supporting HACE1 downregulation in osteosarcoma cells (Fig. 1f). Similar results were obtained by immunostaining of cell pellets for HACE1 expression (data not shown). Next, we assessed *HACE1* transcript levels in two other osteoblast cell lines, OBB and hFOB^43^, and different osteosarcoma cell lines using quantitative reverse transcriptase polymerase chain reaction (RT-PCR), revealing significantly higher *HACE1* expression in osteoblasts compared to osteosarcoma cell lines (*p* < 0.001) (Fig. 1g). This suggests a transcriptional basis for reduced HACE1 expression in osteosarcoma compared to normal osteoblasts. Finally, we assessed HACE1 expression in previously described clonally derived pairs of high and low metastatic osteosarcoma cell lines, namely MG63.3/MG63 and MNNG/HOS cell line pairs^44^. This revealed significantly reduced HACE1 expression in highly metastatic cells of both pairs compared to corresponding low metastatic cells, as assessed by western blotting (Fig. 2a) and immunofluorescence (Fig. 2b; quantified in Fig. 2c). Together, these data reveal reduced HACE1 expression in osteosarcoma, and point to a potential role for HACE1 loss in more aggressive osteosarcoma cells.

### HACE1 expression inhibits the anchorage-independent growth of osteosarcoma cells

**Fig. 3:**
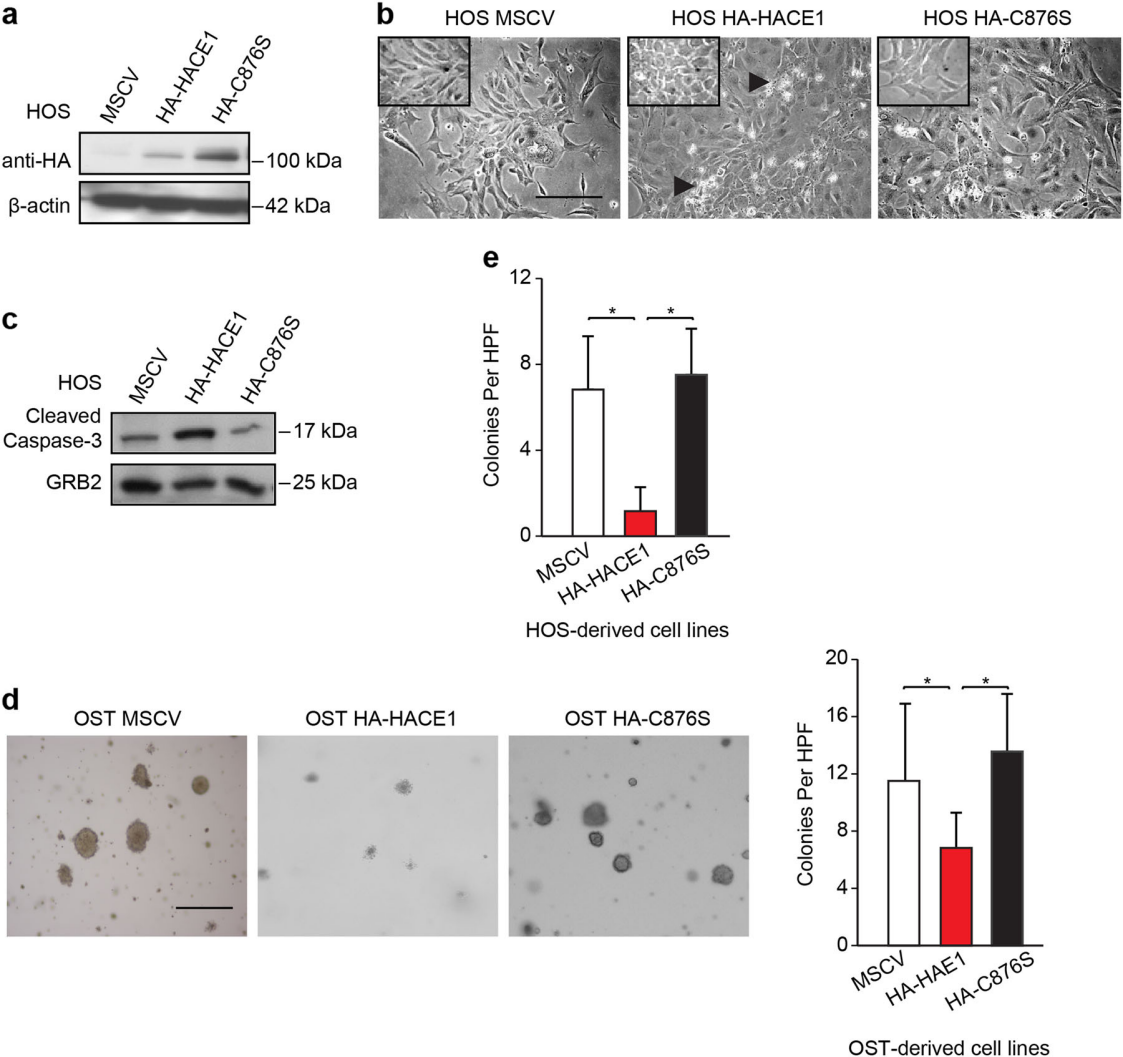
HACE1 mediated inhibitory effects on anchorage-independent osteosarcoma cells colony formation. **a** Immunoblot showing successful transfection of HOS cell line with the indicated vectors as detected by anti-HA antibody. β-Actin was used as a loading control. **b** Effects of functional full-length HA-HACE1 or its inactivated mutant (HA-C876S) overexpression on HOS osteosarcoma cell morphology as detected by phase contrast microscopy. Scale bars: 50 μm. **c** Immunoblot showing the cleaved caspase-3 expression in HOS cells transfected with the indicated vectors. GRB2 was used as a loading control. **d** Left panel: Colony formation in soft agar in the indicated cell lines as detected by phase contrast microscopy 14 days post plating. Right panel: Full-length HACE1 but not its inactivated mutant form; C876S, inhibits cell colony formation in vitro. Colonies were counted under the phase contrast light microscope 14 days after being plated. Results were derived from two independent experiment each in triplicate, and data are presented as mean ± SD. **e** Full-length HACE1 but not its inactivated mutant form, HACE1-C876S, inhibits cell colony formation of HOS cells in vitro, as described in Fig. 3d. In Fig. 3d, e, Mann–Whitney *U* test was used to analyze the data with *p-value < 0.01. MSCV: empty vector control. Scale bars 25 µm

To better understand the role of HACE1 in osteosarcomagenesis, we next assessed effects of ectopic expression of hemagluttinin (HA)-tagged wildtype (*wt*) HACE1 (HA-HACE1) or its ligase inactive mutant, HA-C876S^30^ (see Fig. 3a), on anchorage-independent growth of HOS cells in soft agar, compared to MSCV vector alone. Immunoblotting confirmed successful transfection of the indicated vectors (Fig. 3a). Notably, *wt* HACE1 transfection led to loss of spindle-shaped morphology and acquisition of a more flattened and less refractive cell morphology (Fig. 3b), in addition to enhanced apoptosis as shown by increased cleaved caspase-3 (Fig. 3c). Transfected cells were then plated in soft agar and assessed for colony formation after 14 days of incubation (Fig. 3d). HA-HACE1 expression resulted in marked inhibition of colony formation compared to that of MSCV empty vector or E3 ligase inactive HA-HACE1-C876S-transfected cells (Fig. 3d). In addition, HOS cells transfected with *wt* HACE1 showed significantly reduced soft agar colony formation compared to controls (Fig. 3e; *p* < 0.01). Therefore, ectopic HACE1 expression reduces phenotypic transformation in osteosarcoma cells in vitro.

### HACE1 expression inhibits osteosarcoma cell motility in vitro

**Fig. 4:**
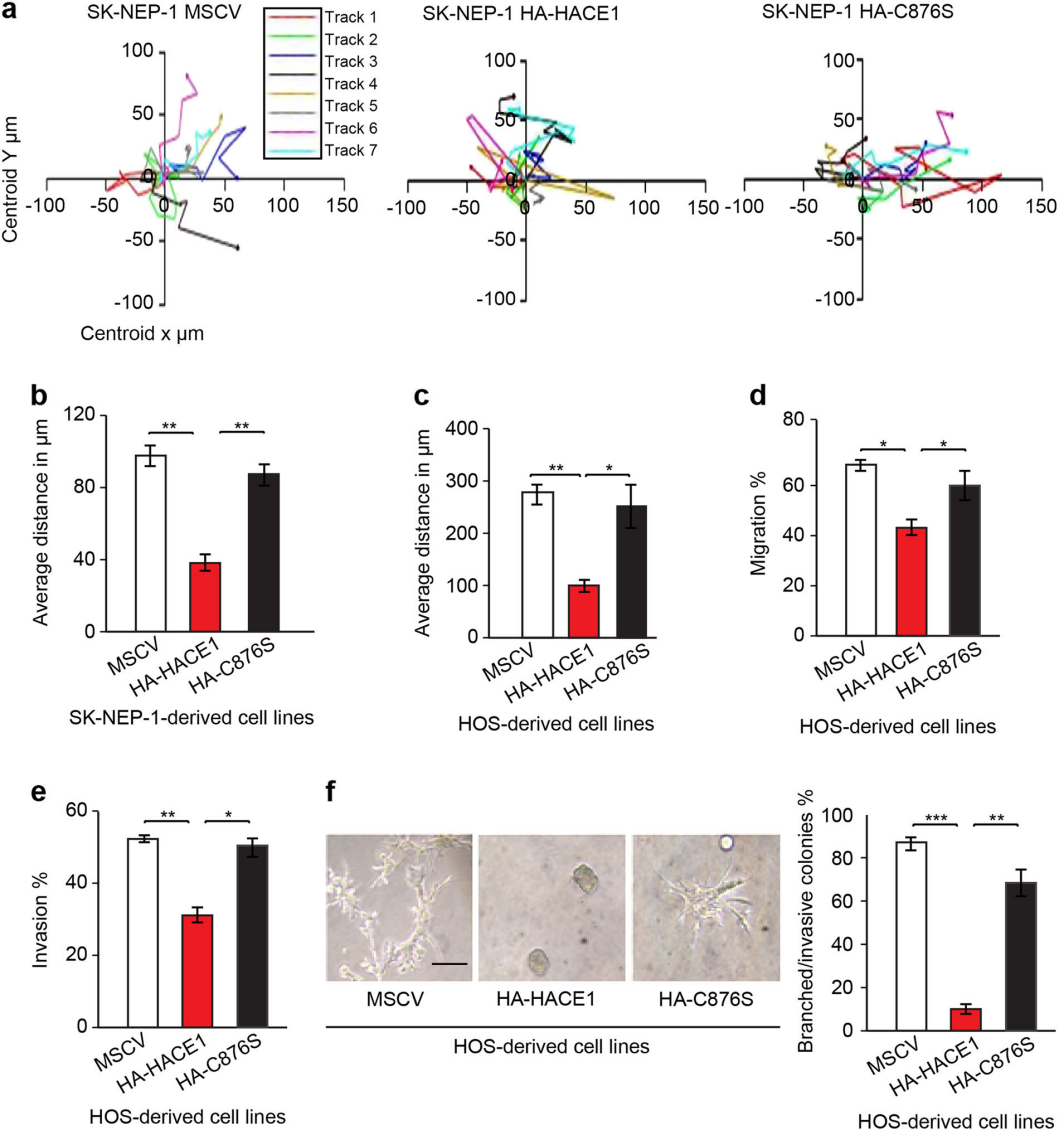
HACE1 inhibits sarcoma cell motility in vitro. **a** Wound-healing assay showing the migration pattern of individual SKNEP1 cells of the indicated cell lines. The migration path of eight cells taken from the wound edge for the indicated cell lines was determined by time-lapse imaging and further analyzed using Volocity software. **b** Charts represent comparison of the average distance traveled +/−SD for the eight cells tracked in each cell line as described in  **a**. **c** Wound-healing assay of the indicated groups of HOS cells. The average distance traveled ±SD for eight cells tracked for each of the indicated cell lines. **d** Boyden chamber trans-well migration assays in which HOS cells were transfected with empty vectors (MSCV), full-length HACE1 (HA-HACE1), or the ligase inactive HA-C876S mutant, and the effects on cell motility were determined by assessing migration of the indicated cells towards serum. **e** In vitro invasion assays showing invasion of the indicated cells through Matrigel towards serum. HOS cells starved for 12 h and plated in 24-well BME-coated chambers for 24 h at 37^o^. Invading cells were then detected by Calcein AM fluorescence using a standard curve. For **d**, **e**, the results of two independent experiments counted in triplicate are represented as a mean ± SD. **f** Left panel: The indicated cell lines were grown on Matrigel for 10 days. Three-dimensional structures were photographed by phase contrast microscopy. MSCV and HACE1 inactivated mutant form; C876S showed highly branched colonies contrary to rounded colonies observed with full-length HACE1. Right panel: quantitation of branched colonies was determined in 10 high-power fields (HPFs) and the average number was represented ± SEM. Scale bars 25 µm. Student's *t*-test was used to analyze the data with *p < 0.01, **p < 0.001

HACE1 loss or silencing is implicated in tumor invasion and metastasis^34,40,45,46^, and so we wondered whether HACE1 might influence osteosarcoma cell motility. We first compared ectopic expression of wt HACE1 vs HACE1-C876S on the motility of SK-NEP-1 cells, as a model of cells lacking HACE1 expression^30^, using time-lapse imaging of wound healing. Ectopic HA-HACE1 significantly inhibited SK-NEP-1 cell motility compared to MSCV or HA-HACE1-C876S transfected cells (Fig. 4a, b). Similarly, HA-HACE1 also significantly reduced HOS cell average distance of migration compared to MSCV or HA-HACE1-C876S-expressing cells in wound-healing assays (Fig. 4c). Moreover, serum-starved HOS cells expressing MSCV or HA-HACE1-C876S displayed marked migration towards serum, which was significantly inhibited by HA-HACE1 expression in the same cells **(**Fig. 4d**)**. In vitro invasion assays revealed significantly reduced invasive capacity of HA-HACE1 expressing HOS cells compared to controls (Fig. 4e). Finally, growth of sarcoma cells in Matrigel three-dimensional cultures showed a clear branching phenotype of MSCV and HA-HACE1-C876S expressing cells, while there was virtually no branching of HA-HACE1-expressing cells **(**Fig. 4f; quantified in the left panel**)**. Indeed, HA-HACE1 transfected cells survived poorly in Matrigel and formed very small, compact round colonies. Taken together, these data show that HACE1 inhibits osteosarcoma cell motility and invasion in vitro.

### HACE1 reduces oxidative stress in osteosarcoma cells and inhibits RAC1 activation

**Fig. 5:**
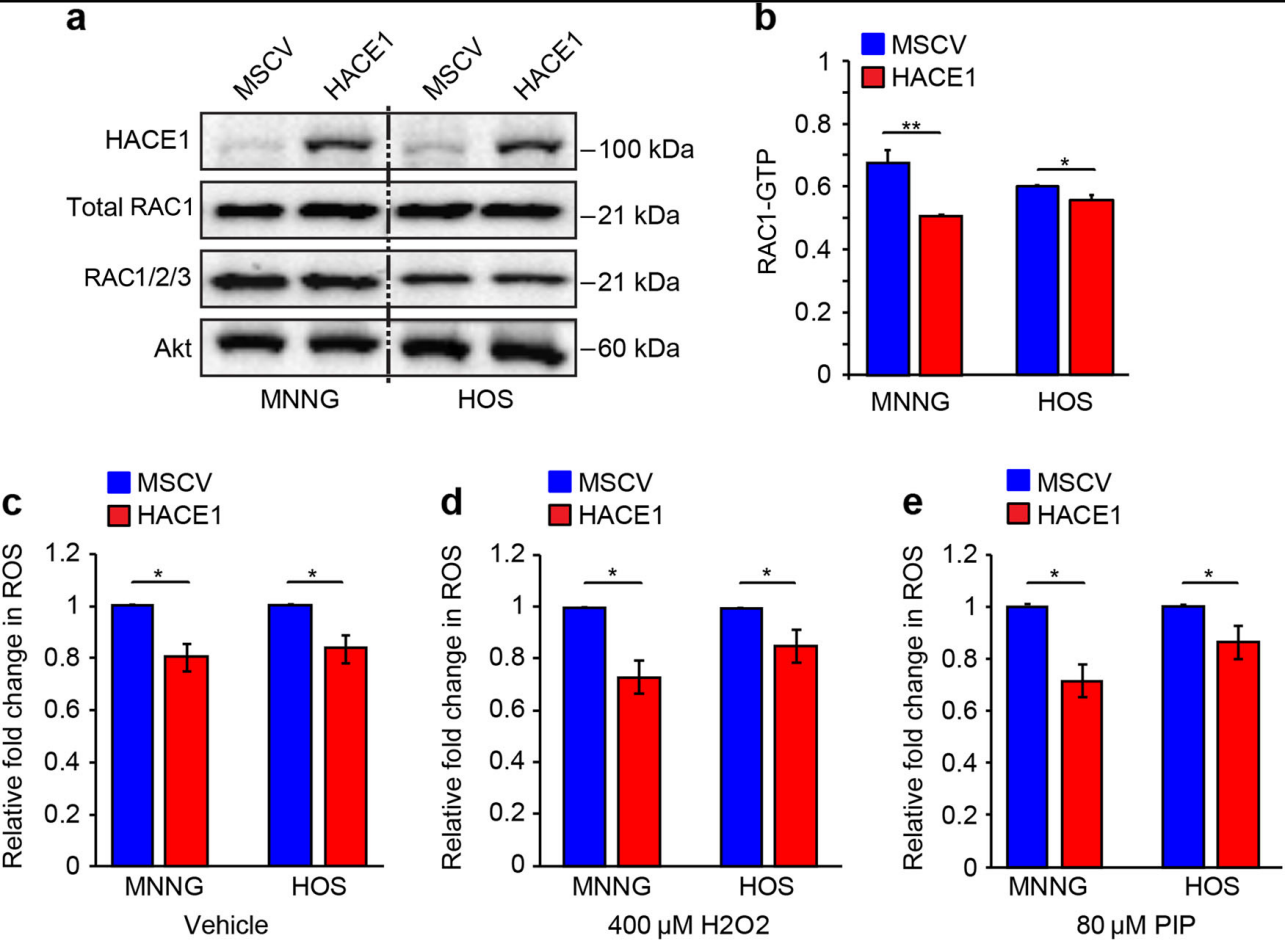
HACE1 reduces activated RAC1 and ROS in osteosarcoma cells in vitro. **a** Immunoblot showing total RAC1 and RAC1/2/3 levels in the indicated cells. Akt was used as a loading control. **b** Active (GTP bound) RAC1 levels as measured by the colorimetric-based G-LISA™ in the indicated cells transfected with empty vector (MSCV) or an HACE1 expression vector. Cells were serum starved overnight, then treated with EGF (10 ng/ml for 30 min). Cell lysates were collected and 25 μg of cell lysates were subjected to the G-LISA™ assay. Absorbance was read at 490 nm. Data were analyzed using a two-tailed Student’s *T*-test, presented as fold change over control; MSCV expressing cells. Error bars indicate SEM for *n* = 3. **c**–**e** The indicated osteosarcoma cells expressing MSCV vector alone or HACE1 were treated with vehicle or 400 µM H_2_O_2_ for 1 h, or with piperlongumine (PIP) 80 µM for 1 h, respectively, as indicated, and then assessed for ROS levels using CM-H2DCFDA. Data were analyzed using a two-tailed Student’s *T*-test, presented as fold change over control MSCV-transfected cells. Error bars indicate SEM for *n* = 3. *p < 0.05, **p < 0.005

HACE1 targets NADPH oxidase-associated RAC1 for ubiquitylation and proteasomal degradation to limit cellular ROS generation^32,34^. To assess if HACE1 also alters RAC1 activity and ROS production in osteosarcoma cells, we first assessed total RAC1 expression in the above MNNG/HOS high/low metastatic cell line pair transfected with vector alone or HA-HACE1. As shown in Fig. 5a, there was no difference in levels of total RAC1, or total RAC (RAC1/2/3), using a pan-RAC antibody, consistent with previous results demonstrating that HACE1 targets activated RAC1, a minor fraction of total cellular RAC1^32^. We next assessed levels of the activated form, RAC1-GTP, in the same cell line pair using G-LISA, a colorimetric-based assay. This showed significant downregulation of RAC1-GTP in HACE1- overexpressing cells, particularly in high metastatic MNNG cells (Fig. 5b). HACE1 overexpression was also associated with reduced ROS levels, as assessed by DCFDA staining (Fig. 5c), in cells treated with ROS-inducing agents, including 400 µM H_2_O_2_ or 80 µM Piperlongamine (PIP) for 1 h (Fig. 5d, e**)**. Together, these support a model whereby HACE1 downregulation results in enhanced RAC1 activation and ROS accumulation, contributing to osteosarcoma progression.

### HACE1 expression inhibits growth and metastatic dissemination of osteosarcoma cell tumor xenografts in immunocompromised mice

**Fig. 6:**
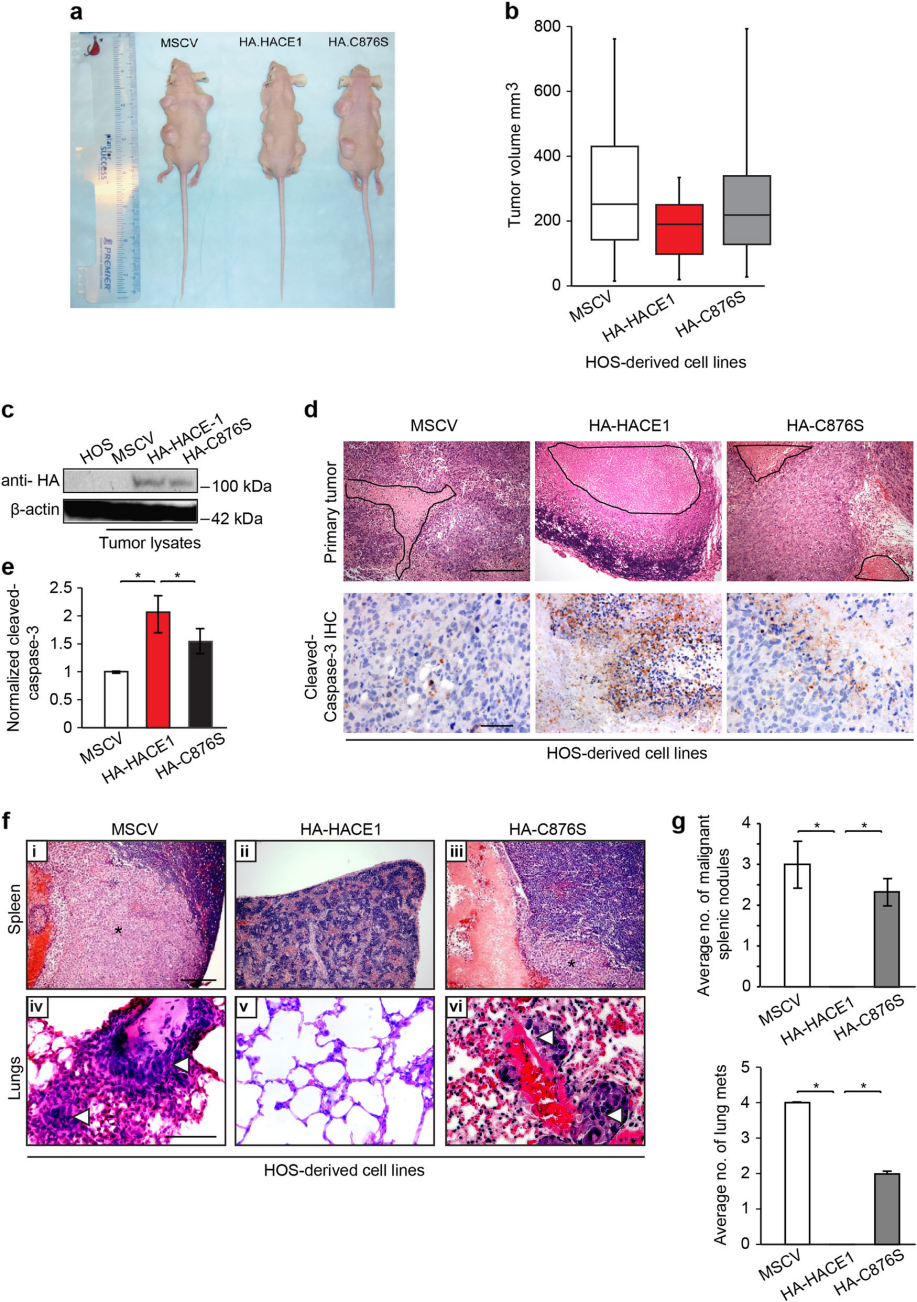
HACE1 inhibits sarcoma cell xenograft growth and metastatic dissemination. **a** Mice inoculated with the indicated HOS-derived cell lines and sacrificed at 27 days post inoculation. **b** Tumor sizes after the 27 days in mice showing significant reduction in the range of the tumor sizes produced by HOS cells expressing full-length HACE1 (HA-HACE1). **c** Immunoblot performed on tissues harvested from the mouse xenograft tumors. β-Actin was used as a loading control. **d** Top panel: H&E staining conducted on the mouse xenograft tumors produced by HOS cells. Black lines demarcate areas of cell death in the indicated groups. Bottom panel: IHC for cleaved caspase-3 conducted on the mouse xenograft tumors produced by HOS cells. **e** Quantitation of cell death (cleaved caspase-3 immunoreactivity) was conducted using ImageJ software and data represented as average value ± SEM for *n* = 15 HPFs in 3 tumors/group. **f** (i–iii) H&E staining conducted on the on the mice spleen sections of the indicated groups. Splenic metastases are denoted by black asterisks; (iv–vi) photomicrographs of H&E staining conducted on the mice lung sections of the indicated groups. Lung metastases are denoted by white arrow heads. **g** Top panel: Average number of splenic metastases (malignant nodules) that developed in mice bearing tumors derived from the indicated osteosarcoma cells. Bottom panel: Average number of lung metastases (mets) developed in mice bearing the indicated osteosarcoma cells. Scale bars 100 µm

To confirm the above HACE1 effects in vivo, HOS osteosarcoma cells expressing MSCV vector alone, HA-HACE1, or HA-HACE1-C876S were injected into the flanks of Nu/Nu Cd-1 male 6-week-old mice (3 mice/group) (see Materials and methods). In vivo growth of osteoarcoma cell lines was monitored, confirming that each cell line readily formed detectable tumors at primary implantation sites (Fig. 6a). HA-HACE1 expressing HOS-derived tumors showed smaller primary implantation site tumor sizes compared to MSCV or HA-HACE1-C876S-derived tumors. Although not statistically significant due to high variability among mice, there was a notable reduction in tumor volumes generated with HA-HACE1 cell lines compared to the others, with a trend towards statistical significance (*p* = 0.05; Fig. 6b). Immunoblot analysis of tumor lysates with anti-HA antibodies confirmed equal HACE1 expression in HA-HACE1 and HA-HACE1-C876S-expressing tumors (Fig. 6c). Histological sections of implantation site tumors revealed extensive areas of necrosis in HA-HACE1 compared to MSCV or HA-HACE1-C876S-expressing tumors (Fig. 6d, upper panels), which was associated with enhanced caspase-3 activity as detected by immunohistochemisry (IHC) (Fig. 6d, lower panel and quantified in Fig. 6e), consistent with its role in enhancing apoptosis^47^. Next, we assessed the ability of HOS cells to metastasize to distant organs. As shown in Fig. 6f, upper panel, quantified in Fig. 6g, upper panel, mice with MSCV and HA-HACE1-C876S-expressing HOS tumors showed marked tumor replacement in spleens from 3/3 tumor-bearing mice, while we failed to detect any tumor spread to splenic tissues of mice bearing HA-HACE1-expressing tumors. We then screened lung sections morphologically for microscopic evidence of lung metastases from the same mice. While an average of four and two metastatic lung lesions were observed in MSCV and HA-HACE1-C876S tumor-bearing mice, respectively, we failed to detect any lung metastases in mice with HA-HACE1-expressing tumors, despite extensive morphologic investigation (Fig. 6f, lower panel quantified in Fig. 6g, lower panel). Therefore HA-HACE1 expression completely eliminated detectable metastases of HOS cells (Fig. 6f, g). Together, these findings provide compelling evidence that HACE1 inhibits growth as well as invasive and metastatic capacity of osteosarcoma cells in vivo.

### Reduced HACE1 expression is associated with poor survival and advanced grade in osteosarcoma

**Fig. 7:**
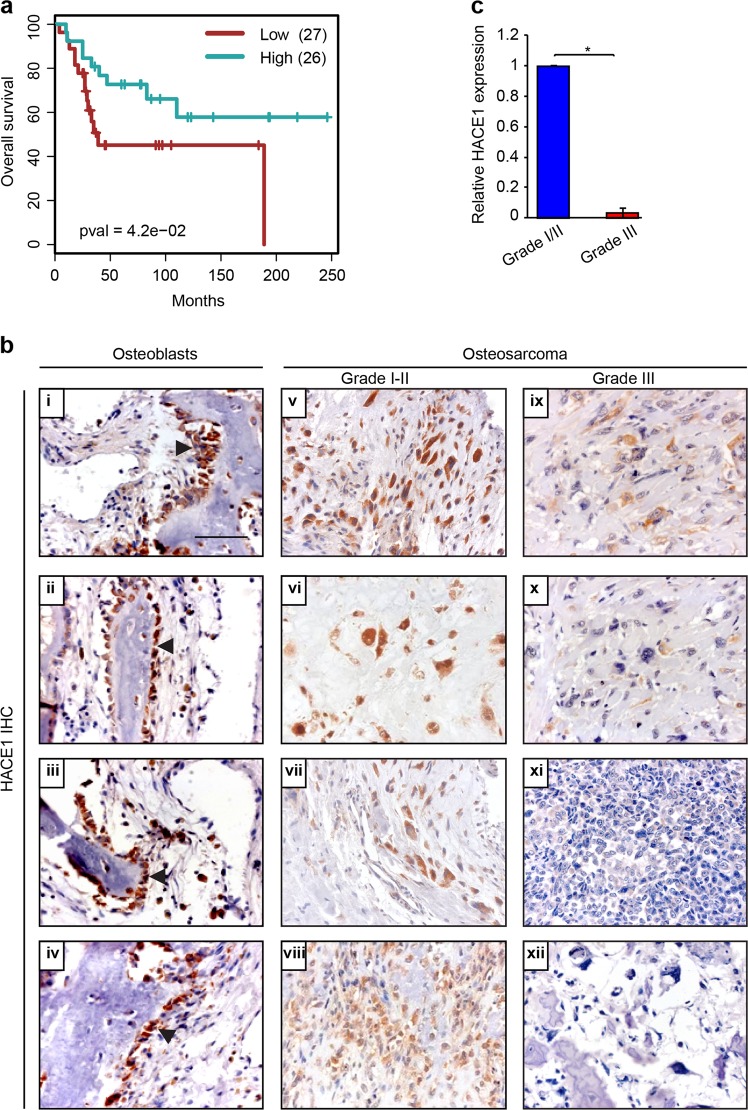
HACE1 downregulation in osteosarcoma is associated with poor outcome and higher osteosarcoma grade. **a** Kaplan Meyer survival curve shows significant (log rank test <0.05) worse overall prognosis for osteosarcoma patients (GSE21257) with low expression levels of HACE1 compared to patients showing high level of the transcript. **b** Examples of HACE1 immunohistochemical staining of osteosarcoma sections. (**i**–**iv**) Strong (+3) nucleo-cytoplasmic HACE1 expression in osteoblasts. Arrows point to osteoblasts. (**v–viii**) Grade I osteosarcomas showing weak (+1)-to-moderate (+2) nucleo-cytoplasmic HACE1 expression. (ix–xii) Grade III osteosarcomas exhibiting weak (+1) occasional cytoplasmic or lack of staining for HACE1. Scale bars, 100 μm. **c** Relative HACE1 expression in grade I/II osteosarcoma (10 cases) vs grade III (15 cases) as measured by a combined immunohistochemistry score (percentage of positive tumor cells × staining intensity) as detailed in the Materials and methods section. Data were analyzed using a two-tailed Student’s *T*-test, and presented as fold change relative to average grade I/II staining. Error bars indicate SEM. *p < 0.05

Finally, we examined *HACE1* expression in a publicly available osteosarcoma database linked to patient outcome (GSE21257), which revealed that lower *HACE1* expression levels significantly correlates with poor overall survival (*p*-value = 4.2e−02) (Fig. 7a). Therefore, HACE1 may serve as a prognostic biomarker in osteosarcoma patients whereby low expression is predictive of poor overall survival. We next performed IHC to assess HACE1 expression in 25 formalin-fixed, paraffin-embedded osteosarcoma cases, selected for the presence of both malignant tissue and adjacent normal bone as an internal control. HACE1 immunoreactivity was estimated by counting the number of positive cells per 1000 tumor cells. Eleven cases (44%) were completely negative for HACE1 staining, while the remaining 14 cases showed variable degrees of HACE1 staining, ranging from positive staining in 5–90% of tumor cells, with a median value of 40%. We therefore performed statistical comparisons of clinicopathological data and HACE1 expression between the two groups (i.e. <40% vs ≥40% HACE1 staining positivity). Low HACE1 expression (i.e. <40% positivity) was observed in 76% of cases and was significantly correlated with high grade (*p*-value = 0.032) and reduced apoptotic counts (*p*-value = 0.01) (Table 1). Representative examples are shown in Fig. 7b, panels i–iv, in which normal bone osteoblasts demonstrated strong nucleo-cytoplasmic staining (i.e. +3; see Materials and methods), compared to weak (+1)-to-moderate (+2) cellular staining observed in Grade I–II osteosarcomas (Fig. 7b, panels v–viii). In contrast, grade III osteosarcomas either showed only very weak staining or were completely negative for HACE1 immunoreactivity (Fig. 7b, panels ix–xii). Collectively, as shown in Fig. 7c, HACE1 expression was dramatically reduced in grade III compared to grade I/II osteosarcoma, further implicating *HACE1* loss in osteosarcoma progression and poor outcome.

**Table 1:** Correlation between HACE1 expression in osteosarcoma cases and clinicopathological parameters

	Osteosarcoma (25 cases)				
Variable	(Less than) <40% (weak staining intensity -to-absent staining) (19/25; 76% of cases)	≥40% (weak staining intensity -to-moderate staining) (6/25; 24% of cases)	Total (25/25; 100%)	Test of significance	*P*-value
**Grade**					
I	3 (12%)	4 (16%)	7 (28%)		
II	2 (8%)	1 (4%)	3 (12%)	*χ*² test	0.032*
III	14 (56%)	1 (4%)	15 (60%)		
Apoptotic count /10 HPFs (±SD)	4.2 ± 2.41	6.6 ± 2.7		T	0.01*

*SD: *standard deviation, *T: *Student’s *t*- test, *χ² test: *Chi-square test

*HPFs:* high power fields

*Significant (*p*  < 0.05)

## Discussion

Osteosarcoma is a genetically complex disease that most often affects children and young adults. The genomic instability observed in osteosarcoma has made efforts to better understand the underlying molecular pathology very challenging. Here we demonstrate reduced expression of the 6q21 *HACE1* tumor suppressor gene in osteosarcoma. *HACE1* inactivation was originally identified in Wilms’ tumor^30^, but is increasingly implicated in other tumor types. In addition to Wilms’ tumor, this includes breast, ovarian, and prostatic carcinomas, leukemias, sarcomas, lymphomas, and natural killer cell neoplasms^30,31,40,41,46^. For example, loss of HACE1 expression plays a critical role in mammary cell transformation and breast cancer progression^40^. In the current study, we hypothesized that *HACE1* loss also contributes to osteosarcoma progression. Compared to normal osteoblasts, both HACE1 protein and transcript levels were significantly reduced in a panel of osteosarcoma cell lines, pointing to transcriptional deregulation of HACE1 in osteosarcoma. Moreover, HACE1 expression in clonally derived pairs of high/low metastatic osteosarcoma cells showed an even further reduction in high compared to low metastatic cells. Further studies are necessary to determine if HACE1 inactivation in osteosarcoma occurs through epigenetic silencing of *HACE1*, as we described for Wilms’ tumor^30,31^.

Previous studies have implicated the 6q21 region, including the *HACE1* locus, in osteosarcoma^41,48^. Tarkannen et al.^49^ examined 31 high-grade osteosarcomas and found losses affecting 6q21–q22 in 32% of specimens. Fletcher et al.^50^ characterized chromosomal abnormalities in 17 high-grade osteosarcomas, and while they found many karyotypic abnormalities, they reported frequent non-random deletions involved 6q21 → qter. Using high-density cDNA microarrays, Squire et al.^51^ showed loss of 6q21 in 3/9 osteosarcoma cases. Further, Ohata et al.^52^ analyzed 12 osteosarcoma cases with comparative genomic hybridization, idenfying highly frequent alterations of chromosome 6q16–23.

Osteosarcoma cells engineered to express *wt HACE1* showed markedly reduced soft agar colony formation and cell motility compared to those expressing ligase dead HACE1-C876S. Moreover, *wt* but not ligase dead HACE1 dramatically reduced branching and invasive morphology of osteosarcoma cells in Matrigel. Therefore, HACE1 tumor suppressor activity in osteosarcoma appears to require E3 ligase activity. HACE1 overexpression inhibited primary tumor growth of osteosarcoma xenografts in vivo, with significantly increased rates of necrosis and caspase-3 activation compared to tumors expressing vector alone or HACE1-C876S in vivo. Therefore, HACE1 loss may enhance tumor survival in vivo. Most dramatically, ectopic of expression of *wt* HACE1 but not the ligase dead mutant led to complete loss of metastatic dissemination of osteosarcoma cells to spleen or lungs. These data highlight the in vivo tumor suppressor activity of HACE1 in osteosarcoma.

Based on publicly available databases, HACE1 expression is significantly downregulated in 76% of osteosarcomas, and this is associated with poor survival. In osteosarcoma clinical specimens, HACE1 protein expression was reduced in malignant cells compared to adjacent normal osteoblasts by IHC. Moreover, we failed to detect HACE1 expression in 11 (44%) of 25 cases analyzed by IHC. In these cases, *HACE1* is likely inactivated genetically or epigenetically. However, in the remaining cases, protein expression was observed in 5–90% of cells, and reduced HACE1 expression (i.e. immunostaining in <40% of cells) significantly correlated with higher tumor grade. In these cases, clonal loss of gene expression is unlikely, and other mechanisms to reduce protein expression, such as altered HACE1 mRNA translation or increased proteasomal degradation, may explain focal loss of expression. Of interest is the altered localization of the HACE1 protein when cases were grouped according to expression levels. Those cases with high expression showed diffuse staining throughout the cell, similar to that observed in normal osteoblasts, while in those cases with low expression, HACE1 protein was localized solely to the cytoplasm and excluded from the nucleus. Although more studies are necessary to elucidate the implications of this observation, one possibility is that in cases retaining low expression, HACE1 may functioning abnormally, either due to HACE1 genetic alterations or changes in other HACE1 pathway components. Therefore HACE1 downregulation was associated with high-grade osteosarcoma, further implicating HACE1 loss in osteosarcoma progression.

HACE1 mitigates ROS generation by targeting RAC1 at RAC1-dependent NADPH oxidases to inhibit superoxide production^34^. HACE1-deficient osteosarcoma cells had elevated  levels of active RAC1, which was associated with increased ROS levels. HACE1 overexpression significantly downregulated both active RAC1 and ROS levels, pointing to a potential link between HACE1 loss and ROS regulation in osteosarcoma. For example, HACE1 loss may enhance ROS accumulation, thus contributing to genomic instability and tumor evolution in osteosarcoma. Alternatively, given that ectopic HACE1 expression only moderately reduced primary xenograft growth but dramatically inhibited dissemination to lungs, HACE1 loss may preferentially benefit tumor cell fitness for metastasis as opposed to primary tumor growth, such as by inactivating HACE1-mediated apoptosis under different stresses of the metastatic cascade.

In summary, these studies highlight HACE1 as a potential tumor suppressor in osteosarcoma. HACE1 inhibits growth, invasion, and metastasis of osteosarcoma cells in vitro and in vivo. Decreased HACE1 expression in osteosarcoma clinical samples is associated with high-grade sarcoma and poor survival. Therefore, HACE1 may serve as a useful prognostic biomarker in osteosarcoma. Future work investigating the potential roles of *HACE1* in osteoblast development and sequencing of the *HACE1* locus in osteosarcoma specimens will be necessary to obtain a better understanding of the functions of this important protein in osteosarcoma.

## Materials and methods

### Cell lines and culture conditions

The immortalized human fibroblast cell line (HF1) was kindly provided by Dr. Peter Lansdorp, University of British Columbia (UBC). The OBB osteoblast line was kindly provided by Dr. Marianne Sadar of UBC. The human osteoblast cell lines OB1 and OB3 were established in our laboratory as previously described^43^. Cells were grown in modified Eagle’s medium (MEM; Gibco) surpplemented with 20% fetal bovine serum (FBS; Gibco) and 1% antibiotic–antimycotic (Invitrogen). Osteogenesis was induced using BMP2/BMP7 heterodimer (R & D Systems, catalog number 3229-BM), and osteoblastic phenotypes were confirmed using Alizarin Red staining, Von Kossa staining and RT-PCR for alkaline phosphatase, collagen type 1A1, bone-specific protein, and osteocalcin as previously described^43^. Osteosarcoma cell lines MNNG, HOS, MG63, and SaOS-2 were obtained from the American Type Culture Collection (ATCC CRL-1547, CRL-1543, CRL-1427, and HTB-85, respectively). OST and SJSA osteosarcoma cell lines were kindly provided by Dr. Tim Triche of Childrens Hospital Los Angeles. The MG63.3 osteosarcoma cell line was kindly provided by Dr. Chand Khanna, and previously described and characterized^53^. Osteosarcoma cell lines were grown in MEM supplemented with 10% FBS and 1% antibiotic–antimycotic. HEK-293 and SK-NEP-1 (ATCC CRL-1573, and ATCC HTB-48, respectively) were used as controls and grown in RPMI supplemented with 10% FBS and 1% antibiotic/antimycotic.

### Generation of HACE1 antibodies

Mouse anti-human HACE1 monoclonal antibody was developed using full-length HACE1 protein as antigen to immunize mice. Mouse serum was then collected and screened with enzyme-linked immunosorbent assay (ELISA). Splenocytes were isolated from the mice with the best immune response and fused with myeloid cells using polyethylene glycol. Fused cells were divided into 96-well plates and cultured with hypoxanthine–aminopterin–thymidine (HAT) medium for at least 5 days. Wells with a single clone growing were identified and the culture supernatants were screened with ELISA. Positive hybridomas were expanded to 24-well plates and then into T-flasks to establish cell lines. Antibody was purified from culture supernatant of hybridoma and confirmed by western blot analysis. Three antibodies were generated, namely 8D3, 3D8, and IES.

### Protein extraction, western blot analysis, and antibodies

Protein extraction and western blotting were carried out using standard protocols^54,55^. β-Actin detection using a rabbit mAb (Cat# 8457 (D7A8); Cell Signaling), GRB2 (Cat# 610111; BD Transduction Laboratories), GAPDH (Cat# 2118; Cell Signaling), or Akt (Cat# 4691; Cell Signaling) were used as loading control. Hemagglutinin (HA) epitope tag monoclonal antibody (anti-HA antibody, Cat# MMS-101P-1000 (1.0 ml), Covance (Cedarlane)) recognizing an HA-epitope tagged HACE1 protein and anti-HACE1 antibody (Cat# ab133637; Abcam), that detect different HACE1 fragments, were used at 1:1000 dilution. Western blotting for total RAC1 was conducted using an anti-RAC1 antibody (Cat# 610650; BD Biosciences), and RAC1/2/3 was assessed using an anti-pan-Rac antibody (Cat# 2465; Cell Signaling). All antibodies were used at a dilution of 1:1000 unless otherwise stated.

### RNA isolation and qRT-PCR

RNA isolation was performed as previously described^56^. Quantitative RT-PCR (qRT-PCR) was performed to assess *HACE1* mRNA levels. The *HACE1* primer/probe set (forward: TCTTA CAGTT TGTTA CGGGC AGTT, probe: [6FAM]CAAAC CCACCATGTG GGACC CTG[TAMRA], reverse: CAATC CACTT CCACC CATGAT) was multiplexed with the VIC-MGB labeled ACTB endogenous control primer/probe kit (Applied Biosystems). Both probe/primer sets were designed to cross exon boundaries, obviating the need for DNAase treatment of the source RNA. Reactions were performed in quadruplicate, and each experiment repeated twice. The reactions were run in an ABI 7000 sequence detection system (Applied Biosystems). The relative expression level of HACE1 was determined using the 2^−ΔΔCT^ analysis method^57^.

### *HA-HACE1* transfection of osteosarcoma cells

Stable *HACE1* re-expression in osteosarcoma cell lines was conducted using a retroviral system. *HACE1* was tagged with haemagglutinin (HA) and transferred into an MSCV vector carrying resistance to hygromycin. The HECT domain cysteine residue, C876, critical for *HACE1* E3 ligase activity^30^, was subjected to site-directed mutagenesis, converting cysteine-876 to serine (C876S) to create a non-functional control. Thus, three vectors were used, firstly a control MSCV empty vector, secondly an MSCV vector containing functional *HA-HACE1* (HA-HACE1 vector), and thirdly an MSCV vector containing the putatively non-functional *HA-C876S* (HA-C876S vector). All vectors used in the current study were generated as previously described^38^.

### Anchorage-independent growth assays

Anchorage-independent cell growth using soft agar colony assays was performed as previously described^58^. Briefly, the bottom layer was constructed using 0.4% agar in DMEM/FBS. Then, cells suspended in 0.25% agar in DMEM/FBS were plated at a concentration of 8000 cells/well and incubated at 37 °C. Single cells and colonies per high-power field were counted and results formulated as the percentage of macroscopic (>0.1 mm) colonies formed/total number of cells plated. The experiment was conducted two times in triplicate for each cell line.

### Matrigel three-dimensional growth assays

Matrigel growth assays were conducted in a six-well plate as previously described^59^. In brief, a 500 µl layer of growth factor reduced Matrigel (BD Biosciences, cat# 354230) was applied to wells and allowed to solidify at 37 °C, generating a bottom layer. Cells were prepared and added slowly on the top of the bottom layer in a final concentration of 20,000 cells/ml. Cultures were assessed to ensure single suspensions and to exclude clump formation. Cells were then fed with 3–4 drops of assay medium containing 2% Matrigel every 3–5 days. The cultures were examined with phase contrast microscopy every 2 days and imaged for phenotypic changes. The experiment was conducted two times in triplicate for each cell line.

### In vitro invasion assays

Assays for cell invasion through the basement membrane using Culturex Coated® 24-Well BME-Coated Cell Invasion platform (Cat# 3480-024-k, Cedarlane) were carried out as previously described^60^.

### Immunohistochemistry

Twenty-five formalin-fixed, paraffin-embedded osteosarcoma cases were identified and stained for HACE1 using 8D3 antibodies, at a dilution of 1:100. For IHC evaluation, the percentages (%) of cells positively staining for HACE1 as well as staining intensity were evaluated in at least 1000 tumor cells. For percentages of positive cells, staining patterns of tumor cells were divided into two categories: (1) positive (whether cytoplasmic, nuclear, or nucleo-cytoplasmic) and (2) negative (with no detectable staining). For staining intensity, we used a 4-point scale (0–3+) as previously described^61^. Tumors with <40% positive cells showed weak (+1) staining intensity or no staining at all, while tumors with ≥40% positive cells showed weak (+1)-to-moderate (+2) staining intensity. All cases were scored by PHS and AME, board-certified pathologists, in a blinded fashion.

### Indirect immunofluorescence of cultured cells and immunostaining of cell pellets

For indirect immunofluorescence, cells were grown on glass slides, and then fixed with 4% formaldehyde and permeated with 0.1% Triton X-100 for 15 min. Subsequent stains and imaging for detection of HACE1 were conducted as previously described^62^. For immunostaining of cell pellets, cells were grown in regular media, and then collected, pelleted, and fixed overnight in 4% buffered formalin with subsequent dehydration in ethanol, and treated with xylene. Cell pellets were then embedded in paraffin and processed for immunostaining as previously described^62^. Image acquisition was conducted using either an Axioplan2 fluorescence microscope (Zeiss) or a Zeiss LSM 780 confocal microscope (Carl Zeiss, Thornwood, NY).

### ROS measurements

Intracellular ROS was determined using the ROS-sensitive probe CM-H2DCFDA (Cat# C6827; Invitrogen). HOS and MNNG cells, transfected with empty vector (MSCV) or full-length HACE1 (HA. HACE1), were seeded in 96-well microplates at a final concentration of 1 × 10^5^ cells per well. Then, cells were treated with vehicle control or ROS-inducing agents, including 400 µM H_2_O_2_ or 80 µM Piperlongamine (PIP) for 1 h. Cells were then incubated with 20 μM DCFDA for 30 min, at 37 °C in the dark. Fluorescent DCFDA signals detected using a mircoplate reader (SpectraMax) at Ex/Em = 495/529 nm.

### RAC1 G-LISA activation assay

The G-LISA RAC1 activation luminescence-based kit (Cat# BK126; Cytoskeleton), previously described^63^, was used to determine active (GTP bound) RAC1 levels in HOS and MNNG cells transfected with empty vector (MSCV) or full-length HACE1 (HA-HACE1), according to the manufacturer’s protocol. In brief, 25 μg total of protein was added to each corresponding well pre-coated with RAC-GTP-binding protein. This was incubated at 4 °C for 30 min followed by successive incubation with 50 μl of anti-RAC1 (1/50 in Antibody Dilution Buffer) for 45 min at RT. Then, secondary antibody conjugated with HRP (1/100 in Antibody Dilution Buffer) was added and incubated for 45 min. Subsequently, 50 μl of HRP detection reagent was added to each well, followed by incubation for another 20 min. The reaction was stopped by the addition of 50 μl HRP stop solution and the absorbance was recorded at 490 nm.

### Mouse tumor xenograft studies

HOS osteosarcoma cell lines stably transfected with empty MSCV vector or vectors encoding HA-HACE1 or HA-C876S were grown to 80% confluence in T-75 flasks and then harvested with trypsin, washed, and suspended at 10^6^ cells/ml. Nu/Nu Cd-1 male 6-week-old mice (3 mice/group) were anaesthetized with isoflurane and injected with 100 μl of cell suspension into each flank, at four sites per mouse. Three mice were used for each of the cell lines, with 12 injections per cell line. Tumors were measured in three orthogonal planes and volumes estimated assuming roughly spherical growth by the formula 0.5236 × length × width × height ((4/3) *π*) (length/2)(width/2) (height/2)), as described by Bogden^64^. On day 27 the mice were euthanized using a CO_2_ chamber and the tumors dissected and weighed. After sacrificing the animals, the primary tumors as well as different organs were collected, and fixed in formalin. Sectioning of the tissues were performed and subsequently processed into paraffin blocks. Histological slides obtained from the paraffin blocks were stained with hematoxylin & eosin; stains were obtained for microscopic evaluation using an Axioplan2 fluorescence microscope (Zeiss). Tumor dissemination to the lung was a sure sign of metastatic spread. Immunohistochemical expression of active caspase-3 was conducted using Abcam antibody, Cat# ab2302 and images were analyzed using ImageJ software.

### Statistical analysis

All statistical analyses were conducted using a Student’s two-tailed *T*-test, unless otherwise indicated, with *p*-values < 0.05 being considered as being statistically significant.

### Microarray data and survival data analyses

Gene expression microarray data for *HACE1* expression was retrieved from the GSE28974 publicly available database for MSCs, and GSE33382 for osteosarcoma samples. Data were processed as described in Kuijjer et al.^65^, and median centered. For Kaplan Meyer survival curves, gene expression microarray data were obtained from GSE21257, and processed as described in Buddingh et al.^66^ and sequentially median centered. All samples with *HACE1* expression lower than or equal to the median were labeled “Low”, and the remainder as “High”. For chromosomal aberrations we re-analyzed publicly available Cytoscan High-Density array data on 113 primary ostoeosarcomas (E-MTAB-4815; https://www.ebi.ac.uk/arrayexpress/experiments/E-MTAB-4815/)^42^. CEL files were processed using the rawcopy R package^67^, and copy number loss/gain thresholds were set as first and third quartiles of the whole-genome segment mean distribution.

## References

[1] Tang N, Song WX, Luo J, Haydon RC, He TC (2008). Osteosarcoma development and stem cell differentiation. Clin. Orthop. Relat. Res..

[2] Marina N, Gebhardt M, Teot L, Gorlick R (2004). Biology and therapeutic advances for pediatric osteosarcoma. Oncologist.

[3] Sandberg AA, Bridge JA (2003). Updates on the cytogenetics and molecular genetics of bone and soft tissue tumors: osteosarcoma and related tumors. Cancer Genet. Cytogenet..

[4] Dorfman HD, Czerniak B (1995). Bone cancers. Cancer.

[5] Gorlick R (2003). Biology of childhood osteogenic sarcoma and potential targets for therapeutic development: meeting summary. Clin. Cancer Res..

[6] Durfee RA, Mohammed M, Luu HH (2016). Review of osteosarcoma and current management. Rheumatol. Ther..

[7] Geller DS, Gorlick R (2010). Osteosarcoma: a review of diagnosis, management, and treatment strategies. Clin. Adv. Hematol. Oncol..

[8] Garwicz S (2000). Second malignant neoplasms after cancer in childhood and adolescence: a population-based case-control study in the 5 Nordic countries. The Nordic Society for Pediatric Hematology and Oncology. The Association of the Nordic Cancer Registries. Int. J. Cancer.

[9] Mirabello L, Troisi RJ, Savage SA (2009). International osteosarcoma incidence patterns in children and adolescents, middle ages and elderly persons. Int. J. Cancer.

[10] Ottaviani G, Jaffe N (2009). The etiology of osteosarcoma. Cancer Treat. Res..

[11] Martin JW, Squire JA, Zielenska M (2012). The genetics of osteosarcoma. Sarcoma.

[12] Overholtzer M (2003). The presence of p53 mutations in human osteosarcomas correlates with high levels of genomic instability. Proc. Natl. Acad. Sci. USA.

[13] Levine AJ, Oren M (2009). The first 30 years ofp53: growing ever more complex. Nat. Rev. Cancer.

[14] Siddiqui R (2005). The TP53 mutational spectrum and frequency of CHEK2*1100delC in Li-Fraumeni-like kindreds. Fam. Cancer.

[15] Castresana JS (1995). Detection of TP53 gene mutations in human sarcomas. Eur. J. Cancer.

[16] Lonardo F, Ueda T, Huvos AG, Healey J, Ladanyi M (1997). p53 and MDM2 alterations in osteosarcomas: correlation with clinicopathologic features and proliferative rate. Cancer.

[17] Bougeard G (2015). Revisiting Li-Fraumeni syndrome from TP53 mutation carriers. J. Clin. Oncol..

[18] Chen, Z., Guo, J., Zhang, K. & Guo, Y. TP53 mutations and survival in osteosarcoma patients: a meta-analysis of published data. Dis. Markers **2016**, 4639575 (2016). 10.1155/2016/4639575 PMC4863100 27239089

[19] Gokgoz N (2001). Comparison of p53 mutations in patients with localized osteosarcoma and metastatic osteosarcoma. Cancer.

[20] Tsuchiya T (2000). Analysis of the p16INK4, p14ARF, p15, TP53, and MDM2 genes and their prognostic implications in osteosarcoma and Ewing sarcoma. Cancer Genet. Cytogenet..

[21] Dimaras H (2015). Retinoblastoma. Nat. Rev. Dis. Primers.

[22] Scott MC (2015). Aberrant retinoblastoma (RB)-E2F transcriptional regulation defines molecular phenotypes of osteosarcoma. J. Biol. Chem..

[23] Feugeas O (1996). Loss of heterozygosity of the RB gene is a poor prognostic factor in patients with osteosarcoma. J. Clin. Oncol..

[24] Wang LL (2003). Association between osteosarcoma and deleterious mutations in the RECQL4 gene in Rothmund-Thomson syndrome. J. Natl. Cancer Inst..

[25] Mohaghegh P, Hickson ID (2001). DNA helicase deficiencies associated with cancer predisposition and premature ageing disorders. Hum. Mol. Genet..

[26] Luo T, Yi X, Si W (2017). Identification of miRNA and genes involving in osteosarcoma by comprehensive analysis of microRNA and copy number variation data. Oncol. Lett..

[27] Chiappetta C (2017). Whole-exome analysis in osteosarcoma to identify a personalized therapy. Oncotarget.

[28] Chen X (2014). Recurrent somatic structural variations contribute to tumorigenesis in pediatric osteosarcoma. Cell Rep..

[29] Kovac M (2015). Exome sequencing of osteosarcoma reveals mutation signatures reminiscent of BRCA deficiency. Nat. Commun..

[30] Anglesio MS (2004). Differential expression of a novel ankyrin containing E3 ubiquitin-protein ligase, Hace1, in sporadic Wilms’ tumor versus normal kidney. Hum. Mol. Genet..

[31] Zhang L (2007). The E3 ligase HACE1 is a critical chromosome 6q21 tumor suppressor involved in multiple cancers. Nat. Med..

[32] Torrino S (2011). The E3 ubiquitin-ligase HACE1 catalyzes the ubiquitylation of active Rac1. Dev. Cell.

[33] Castillo-Lluva S, Tan CT, Daugaard M, Sorensen PH, Malliri A (2013). The tumour suppressor HACE1 controls cell migration by regulating Rac1 degradation. Oncogene.

[34] Daugaard M (2013). Hace1 controls ROS generation of vertebrate Rac1-dependent NADPH oxidase complexes. Nat. Commun..

[35] Acosta MI (2018). Group-I PAKs-mediated phosphorylation of HACE1 at serine 385 regulates its oligomerization state and Rac1 ubiquitination. Sci. Rep..

[36] Tortola L (2016). The tumor suppressor Hace1 is a critical regulator of TNFR1-mediated cell fate. Cell Rep..

[37] Liu Z (2014). Ubiquitylation of autophagy receptor Optineurin by HACE1 activates selective autophagy for tumor suppression. Cancer Cell..

[38] Zhang L (2014). HACE1-dependent protein degradation provides cardiac protection in response to haemodynamic stress. Nat. Commun..

[39] Tang D (2011). The ubiquitin ligase HACE1 regulates Golgi membrane dynamics during the cell cycle. Nat. Commun..

[40] Goka ET, Lippman ME (2015). Loss of the E3 ubiquitin ligase HACE1 results in enhanced Rac1 signaling contributing to breast cancer progression. Oncogene.

[41] Hoogerwerf WA, Hawkins AL, Perlman EJ, Griffin CA (1994). Chromosome analysis of nine osteosarcomas. Genes Chromosomes Cancer.

[42] Smida J (2017). Genome-wide analysis of somatic copy number alterations and chromosomal breakages in osteosarcoma. Int. J. Cancer.

[43] El Naggar A (2012). Expression and stability of hypoxia inducible factor 1alpha in osteosarcoma. Pediatr. Blood Cancer.

[44] Ren L (2015). Characterization of the metastatic phenotype of a panel of established osteosarcoma cells. Oncotarget.

[45] Gao ZF (2016). Tumor-suppressive role of HACE1 in hepatocellular carcinoma and its clinical significance. Oncol. Rep..

[46] Kucuk C (2013). HACE1 is a tumor suppressor gene candidate in natural killer cell neoplasms. Am. J. Pathol..

[47] Tortola L (2016). The tumor suppressor Hace1 is a critical regulator of TNFR1-mediated cell fate. Cell Rep..

[48] Bridge JA (1997). Cytogenetic findings in 73 osteosarcoma specimens and a review of the literature. Cancer Genet. Cytogenet..

[49] Tarkkanen M (1999). DNA sequence copy number increase at 8q: a potential new prognostic marker in high-grade osteosarcoma. Int. J. Cancer.

[50] Fletcher JA, Gebhardt MC, Kozakewich HP (1994). Cytogenetic aberrations in osteosarcomas. Nonrandom deletions, rings, and double-minute chromosomes. Cancer Genet. Cytogenet..

[51] Squire JA (2003). High-resolution mapping of amplifications and deletions in pediatric osteosarcoma by use of CGH analysis of cDNA microarrays. Genes Chromosomes Cancer.

[52] Ohata N (2006). Highly frequent allelic loss of chromosome 6q16-23 in osteosarcoma: involvement of cyclin C in osteosarcoma. Int. J. Mol. Med..

[53] Khanna C (2000). An orthotopic model of murine osteosarcoma with clonally related variants differing in pulmonary metastatic potential. Clin. Exp. Metastasis.

[54] Sorokin AV (2005). Proteasome-mediated cleavage of the Y-box-binding protein 1 is linked to DNA-damage stress response. EMBO J..

[55] El-Naggar AM (2015). Translational activation of HIF1alpha by YB-1 promotes sarcoma metastasis. Cancer Cell..

[56] Clegg NJ (2011). MYC cooperates with AKT in prostate tumorigenesis and alters sensitivity to mTOR inhibitors. PLoS ONE.

[57] Livak KJ, Schmittgen TD (2001). Analysis of relative gene expression data using real-time quantitative PCR and the 2(-Delta Delta C(T)) Method. Methods.

[58] Poczobutt JM, Tentler J, Lu X, Schedin PJ, Gutierrez-Hartmann A (2010). Benign mammary epithelial cells enhance the transformed phenotype of human breast cancer cells. BMC Cancer.

[59] Debnath J, Muthuswamy SK, Brugge JS (2003). Morphogenesis and oncogenesis of MCF-10A mammary epithelial acini grown in three-dimensional basement membrane cultures. Methods.

[60] Liu X (2009). MicroRNA-222 regulates cell invasion by targeting matrix metalloproteinase 1 (MMP1) and manganese superoxide dismutase 2 (SOD2) in tongue squamous cell carcinoma cell lines. Cancer Genomics Proteomics.

[61] Koo CL (2009). Scoring mechanisms of p16INK4a immunohistochemistry based on either independent nucleic stain or mixed cytoplasmic with nucleic expression can significantly signal to distinguish between endocervical and endometrial adenocarcinomas in a tissue microarray study. J. Transl. Med..

[62] Ioannou M (2010). Validated analysis of HIF-1alpha expression in cancer cells using a controlled and comparative immunoassay. Oncol. Rep..

[63] Hayashida T, Jones JC, Lee CK, Schnaper HW (2010). Loss of beta1-integrin enhances TGF-beta1-induced collagen expression in epithelial cells via increased alphavbeta3-integrin and Rac1 activity. J. Biol. Chem..

[64] Bogden AE, Kelton DE, Cobb WR, Gulkin TA, Johnson RK (1978). Effect of serial passage in nude athymic mice on the growth characteristics and chemotherapy responsiveness of 13762 and R3230AC mammary tumor xenografts. Cancer Res..

[65] Kuijjer ML (2012). Identification of osteosarcoma driver genes by integrative analysis of copy number and gene expression data. Genes Chromosomes Cancer.

[66] Buddingh EP (2011). Tumor-infiltrating macrophages are associated with metastasis suppression in high-grade osteosarcoma: a rationale for treatment with macrophage activating agents. Clin. Cancer Res..

[67] Mayrhofer M, Viklund B, Isaksson A (2016). Rawcopy: improved copy number analysis with Affymetrix arrays. Sci. Rep..

